# Characteristics, hospital referrals and 60-day mortality of older patients living in nursing homes with COVID-19 assessed by a liaison geriatric team during the first wave: a research article

**DOI:** 10.1186/s12877-021-02565-4

**Published:** 2021-10-29

**Authors:** Lorena García-Cabrera, Noelia Pérez-Abascal, Beatriz Montero-Errasquín, Lourdes Rexach Cano, Jesús Mateos-Nozal, Alfonso Cruz-Jentoft

**Affiliations:** 1 Unidad de Cuidados Paliativos, Hospital Universitario Ramón y Cajal (IRYCIS), Carretera de Colmenar km 9,1, 28034 Madrid, Spain; 2 Servicio de Geriatría. Hospital Universitario Ramón y Cajal (IRYCIS), Madrid, Spain

**Keywords:** COVID-19, Mortality, Nursing homes, Liaison geriatric team

## Abstract

**Background:**

The infection by SARS-CoV-2 (COVID-19) has been especially serious in older patients. The aim of this study is to describe baseline and clinical characteristics, hospital referrals, 60-day mortality, factors associated with hospital referrals and mortality in older patients living in nursing homes (NH) with suspected COVID-19.

**Methods:**

A retrospective observational study was performed during March and April 2020 of institutionalized patients assessed by a liaison geriatric hospital-based team. Were collected all older patients living in 31 nursing homes of a public hospital catchment area assessed by a liaison geriatric team due to the suspicion of COVID-19 during the first wave, when the hospital system was collapsed. Sociodemographic variables, comprehensive geriatric assessment, clinical characteristics, treatment received including care setting, and 60-days mortality were recorded from electronic medical records. A logistic regression analysis was performed to analyze the factors associated with mortality.

**Results:**

419 patients were included in the study (median age 89 years old, 71.6 % women, 63.7 % with moderate-severe dependence, and 43.8 % with advanced dementia). 31.1 % were referred to the emergency department in the first assessment, with a higher rate of hospital referral in those with better functional and mental status. COVID-19 atypical symptoms like functional decline, delirium, or eating disorders were frequent.

36.9% had died in the 60 days following the first call. According to multivariate logistic regression age (p 0.010), Barthel index <60 (p 0.002), presence of tachypnea (p 0.021), fever (p 0.006) and the use of ceftriaxone (p 0.004) were associated with mortality. No mortality differences were found between those referred to the hospital or cared at the nursing home.

**Conclusions and implications:**

31% of the nursing home patients assessed by a liaison geriatric hospital-based team for COVID-19 were referred to the hospital, being more frequently referred those with a better functional and cognitive situation. The 60-days mortality rate due to COVID-19 was 36.8% and was associated with older age, functional dependence, the presence of tachypnea and fever, and the use of ceftriaxone. Geriatric comprehensive assessment and coordination between NH and the hospital geriatric department teams were crucial.

## Background

The coronavirus disease 2019 (COVID-19) was declared a pandemic on March 11, 2020, by the World Health Organization. The severity of this disease increases with age, with a mortality rate in those over 80 years of age between 20 and 30 %, [1, 2] and deaths in nursing homes (NH) that represent up to 50 % of total mortality, with a huge variability between countries [3, 4].  However, the analysis of 30-days mortality in hospitalized patients over 65 years old seems to be similar to that observed in nursing homes [5–7] and a relationship has been described between mortality of institutionalized patients due to COVID-19 with the male sex, cognitive and physical impairments, frailty, the presence of dyspnea or severe forms of the disease [8].

During the first wave of SARS CoV-2, a liaison geriatric hospital-based team was created in different hospitals of the Madrid region for the assessment of older patients in NH, supporting on site care and treatment, or recommending referral to emergency departments for acute cases when needed [7, 9].  At that time (first wave) the hospital system in Madrid was almost collapsed, with 105 % of the beds occupied by COVID-19 patients [10].

In Spain, it has been calculated 47-50 % of deaths by COVID-19 in the first wave occurred in this population, [4] which has generated a debate on the suitability of healthcare in the NH during the first wave. Recently, a study has been published where 30-day survival rate of institutionalized older patients with COVID-19 did not depend of where they were treated [7].

There are some published information on the clinical differences in institutionalized and COVID-19-infected older persons treated in their NH or referred to the hospital. However, in most of the available studies, either there is no follow-up or this follow-up is restricted to the first 30 days when it is known that the consequences and repercussions of this disease are prolonged in a longer period in many cases.

Therefore, our study aims to describe the clinical characteristics, the hospital referrals, the 60-day mortality, and the factors related including care setting (NH vs. hospital) in institutionalized patients with COVID-19.

## Methods

A retrospective observational study of institutionalized patients with COVID-19 (clinical suspected or confirmed) who required telephone assessment from a liaison geriatric hospital-based team between 30 March and 30 April 2020 was performed. This team assessed any older patients from 31 NH patients between 8 a.m. and 10 p.m. from Monday to Sunday. During this period the hospital had to increase the number of beds from some 750 to a peak of 983, most of them filled by COVID-19 patients, and to increase fourfold the number of ICU beds [11].

We included all patients assessed for COVID-19 clinical suspicion (presence of respiratory symptoms, fever, delirium, functional impairment, decrease or eating disorders, with no alternative diagnosis) or with a confirmation of diagnostic test of active infection (SARS-CoV-2 PCR availability was very limited in the first weeks of March and increased exponentially thereafter). We excluded older patients assessed for other reasons or patients assessed living in disable person centers.

We collected the following variables from clinical records based on the information provided by the NH healthcare professionals: age, sex, nursing home type, Barthel index, Functional Assessment Classification (FAC) for gait, Reisberg’s Global Deterioration Scale for dementia staging (GDS), Charlson index, and malnutrition (BMI <22 in the last year, albumin <3.5 g / dl in the 6 months prior or active prescription of nutritional supplements). Also, we recorded COVID-19 related symptoms and date of onset (cough, dyspnea, falls, delirium, functional impairment related to asthenia, general malaise or deterioration of physical capacity, eating disorder defined as hyporexia, refusal to eat, or inability to use the oral route), as well as signs of COVID-19 severity (respiratory failure defined as oxygen saturation less than 92 %, dyspnea, tachypnea, referred to as more than 30 breaths per minute and a measured fever ≥38ºC), and COVID-19 diagnostic test, prescribed treatments (hydroxychloroquine, lopinavir/ritonavir, antibiotic therapy, corticosteroid therapy, low molecular weight heparin), emergency department referrals, hospital admissions, and 60-day mortality. Hydroxychloroquine was used with the prior verbal consent of the patient or relatives, who were informed of its use outside the technical data specifications.

In patients referred to the hospital after the first telephone assessment, we reviewed the result of the PCR for COVID-19, whether they required hospital admission and the length of hospitalization.

A descriptive analysis was performed with measures of frequency, mean, and deviation using the Chi-square test for qualitative variables and U-Mann -Whitney for quantitative variables with non-normal distribution. Also, a univariate and multivariate logistic regression model was used for 60-day mortality analysis. Significance was established at *p* <0.05. Data analysis was performed using the IBM SPSS Statistics version 20.

 The study was approved by the Ethics Committee for Healthcare (Comité de Ética para la Asistencia Sanitaria, CEAS) of the Hospital (approved June 8, 2020, act 393). Informed consent was waived for this retrospective study. All the ethical principles for medical investigation in human beings recorded in the Helsinki declaration of the World Medical Association were followed.

## Results

511 patients were assessed between March 30 and April 30 with an average number of 1.51 calls per patient and a range between 1 and 8. We excluded 92 patients assessed for other reasons with a final sample of 419. Among our population 196 (46.8 %) had a positive COVID-19 diagnostic test.

### Participant’s characteristics

Patient characteristics are shown on Table 1, and correspond with a usual NH population of extreme old age and severe physical and mental disability. COVID-19 symptoms were mostly respiratory, but included geriatric syndromes as falls, delirium or refusal to eat. The median from symptom onset to telephone consultation was 3 days. The most used treatments were hydroxychloroquine (64.2 %), azithromycin (53.2 %), and ceftriaxone (29.4 %).

**Table 1:** Characteristics of the sample and according to the place of care

Variable	Total (N= 419)	Nursing home (N= 289)	Hospital (N= 130)	p -Value
Median Age (Q1; Q3)	89 (84;92)	88 (83;92)	89 (84.5;93)	0.169
Gender. % Female	71.6	76.8	60	<0.001
Male	28.4	23.2	40	
Type of nursing home. % Private	68.2	68.5	67.7	0.868
Geriatric Assessment. %				
Barthel <60	63.7	73	43.9	< 0.001
FAC <3	54.6	63.1	34.3	< 0.001
GDS 6-7	43.8	54	22.4	< 0.001
Malnutrition	64	62.9	66.3	0.574
Median Charlson (Q1; Q3)	2 (1,3)	3 (1;4)	2 (1;3)	0.415
Median symptom evolution time (Q1; Q3)	3 (1;7)	3 (1;7))	3 (1;6.8)	0.141
Symptoms. %				
Cough	30.1	28.4	33.8	0.258
Respiratory failure (SatO2 ≤ 92%)	70.2	65.1	81.5	< 0.001
Tachypnea (FR ≥ 30 rpm)	18.9	16.6	23.8	0.08
Dyspnea	16.2	12.8	23.8	0.005
Fever (Tª ≥ 38ºC)	44.2	42.6	47.7	0.328
Delirium	19.6	20.4	17.7	0.516
Functional impairment	20	15.6	30	< 0.001
Falls	3.8	1.4	9.2	< 0.001
Eating Disorder	18.6	18.3	19.2	0.828
Treatment %				
Hydroxychloroquine	64.2	59.2	75.4	0.001
Lopinavir/Ritonavir	5	0	16.2	< 0.001
Azithromycin	53.2	48.1	64.6	0.002
Ceftriaxone	29.4	21.1	47.7	< 0.001
Amoxicillin-Clavulanic Acid	25.5	23.5	30	0.16
Other Antibiotics	28.4	20.8	45.4	< 0.001
Corticosteroids	26.7	18	46.2	< 0.001
HBPM	24.1	6.6	63.1	< 0.001
60-day mortality %	36.8	34.9	40.8	0.253
Visits to emergency department 60 days %	22	8.3	20.2	< 0.001
Hospital admissions 60 days %	7.2	4.5	13.2	0.002
Place of Death. % (N 154)				
Nursing home	70.1	98	16.9	< 0.001
Hospitalization ward	24.7	1	69.9	
Emergency department	3.3	1	7.5	
Palliative care unit	1.9	0	5.6	

31 % were referred to the emergency department, 79 % of them were hospitalized with an average length of stay of 9.1 days and an average time of emergency department referral since the onset of the symptoms of 9.3 days.

Hospital mortality was 27.7 % and 60-day mortality was 36.8 %. 70.1 % of deaths happened in the NH, 28 % in the hospital, and 1.9 % in a palliative care center.

### Patient characteristics according to the place of care (Table 1)

Patients referred to the hospital had a better functional and mental status (Barthel<60 43.9 % vs. 73 %, *p* <0.001, FAC<3 34.3 % vs. 63.1 %, *p* <0.001, GDS 6-7 22.4 % vs. 54 %, *p* <0.001). Hospital referral was also associated with male gender (40 % vs. 23.2 %, *p* <0.001), and with the presence of severe symptoms like respiratory failure (81.5 % vs. 65.1 %, *p* <0.001), dyspnea (23.8 % vs. 12.8 %, *p* < 0.001), functional decline (30 % vs. 15.6 %, *p* <0.001) or falls (9.2 % vs. 1.4 %, *p* <0.001).

Patients referred at hospital after the first phone assessment received more frequently the following treatments: hydroxychloroquine (75.4 % vs. 59.2 %, *p* 0.001), azithromycin (64.6 % vs. 48.1 %, *p* 0.002), ceftriaxone (47.7 % vs. 21.1 %, *p* <0.001), other antibiotics (45.4 % vs. 20.8 %, *p* <0.001) corticosteroids (46.2 % vs. 18 %, *p* <0.001) and low molecular weight heparin (63.1 % vs. 6.6 %, *p* <0.001). Those patients who were referred on the first call and returned to the residence were more frequently referred back to the hospital than those who had not been referred initially (13.2 % vs. 4.5 %, *p* 0.002) (Table 1).

No differences in hospital referrals were found in terms of age, comorbidity, type of nursing home, time of evolution from the onset of symptoms, or mortality at 60 days.

### Factors related to 60-day mortality (Table 2)

The mortality at 60 days was 36.8 % and was associated in univariate analysis with functional and mental impairment (Barthel<60 75 % vs. 57.3 %, *p* <0.001; FAC <3 69.4 % vs. 46.8 %, *p* <0.001 and GDS 6-7 50.7 % vs. 39.9 %, *p* 0.026) and presence of malnutrition (72.2 % vs. 59.8 %, *p* 0.026). There was also a relationship with respiratory failure (79.9 % vs. 64.5 %, *p* <0.001), tachypnea (30.5 % vs. 12.1 %, *p* <0.001), fever (57.4 % vs. 36.2 %, *p* <0.001), delirium (25.3 % vs. 16.2 %) and eating disorders (24 % vs. 15.5 %, *p* 0.03).

**Table 2:** Characteristics of the sample according to mortality at 60 days - Univariate analysis

Variable	Alive (N= 265)	Dead (N= 154)	p value
Median Age (Q1; Q3)	88 (84;92)	90 (85;93.3)	0.043
Gender. % Female	72.5	70.1	0.611
Type of nursing home. % private	68.3	68.2	0.980
Geriatric Assessment. %			
Barthel <60	57.3	75	< 0.001
FAC <3	46.8	69.4	< 0.001
GDS 6-7	39.9	50.7	0.026
Malnutrition	59.8	72.2	0.026
Median Charlson (Q1; Q3)	2 (1;3)	2 (1;3.3)	0.129
Median symptom evolution time (days) (Q1; Q3)	3 (1;7)	3 (1;6.8)	0.486
Symptoms. %			
Cough	32.1	26.6	0.241
Respiratory failure (SatO2 ≤ 92%)	64.5	79.9	< 0.001
Tachypnea (FR ≥ 30 rpm)	12.1	30.5	< 0.001
Dyspnea	12.1	23.4	0.002
Fever (Tª ≥ 38ºC)	36.2	57.4	< 0.001
Delirium	16.2	25.3	0.024
Functional impairment	20.4	19.5	0.825
Falls	4.5	2.6	0.32
Eating Disorder	15.5	24	0.030
Referral to emergency department %	29.1	34.3	0.253
Treatment %			
Hydroxychloroquine	64.9	63	0.693
Lopinavir/Ritonavir	4.9	5.2	0.896
Azithromycin	54.3	51.3	0.548
Ceftriaxone	22.6	40.9	< 0.001
Amoxicillin-Clavulanic Acid	25.7	25.3	0.939
Other Antibiotics	27.2	30.5	0.464
Corticosteroids	23.4	32.5	0.043
HBPM	24.9	22.7	0.615

No differences were found in mortality according to comorbidity, hospital referral (34.3 % vs. 29.1 % *p* 0.253), sex, type of nursing home, time of evolution from the onset of symptoms or to the administration of the different treatments except the use of ceftriaxone, which was more frequent among the deceased (40.9 % vs. 22.6 %, *p* <0.001).

In 60-days mortality multivariate analysis, a significant association was observed with age [OR 1.05 (CI 1.01-1.09) *p* 0.010], functional dependence measured as Barthel <60 [OR 2.57 (CI 1.4-4.7) *p* 0.002], tachypnea [OR 2.12 (CI 1.12-4.01) *p* 0.021], fever [OR 1.9 (CI 1.21-3.21) *p* 0.006] and the use of ceftriaxone [OR 2.2 (CI 1.28-3.78), *p* 0.004] Table 3.

**Table 3:** Multiple logistic regressions analysis* of 60-day mortality

Variable	OR	95% CI	p -Value
Age (by years)	1.05	1.01-1.09	0.010
Barthel Index <60	2.57	1.4-4.7	0.002
Tachypnea	2.12	1.12-4.01	0.021
Fever	1.9	1.21-3.21	0.006
Ceftriaxone	2.2	1.28-3.78	0.004

*The variables included in the multivariate analysis were: age, sex, Barthel, GDS, respiratory failure, tachypnea, dyspnea, fever, delirium, eating disorder, use of ceftriaxone, corticosteroids, and hospital referral

## Discussion

The population referred has a high age, a high dependency rate, dementia, comorbidity, and malnutrition, and those conditions are similar to those described by other authors in similar populations [12–14]. Furthermore, we found a high prevalence of COVID-19 atypical symptoms such as delirium, functional impairment, or eating disorder with percentages around 20 %. Those percentages were similar to other studies in institutionalized patients with COVID-19 [6, 15].

Almost a third of the assessed patients were referred to the hospital. These referrals were associated with a better functional and cognitive situation, the presence of severe symptoms, and the prescription of a specific treatment. A higher hospital referral was also observed in male patients in contrast to the data from Bielza [7]. Therefore, we analyzed characteristics based on gender, observing that institutionalized men with COVID-19 were younger and suffered less functional or cognitive impairment, which could justify this finding.

Additionally, referred patients to the hospital presented serious signs and symptoms of SARS-CoV-2 infection such as respiratory failure, fever, or tachypnea, also described in other studies [7, 8, 12]. The in-hospital mortality rate was 27.7 %, similar to other studies of hospitalized older patients with percentages between 27 % and 49 % [16, 17].

Few studies assess mortality at two months in institutionalized patients with COVID-19. In a study carried out in Italy, there was an excess of mortality observed in the months of March and April 2020 in a nursing home, with a mortality of 43 % among patients with COVID-19 [18]. The mortality observed in our population at 60 days was 36.8 %, a percentage similar to that described in other studies that evaluated mortality at 30 days, finding a rate between 20 and 48 % in institutionalized older persons patients [8, 15, 19, 20].

The mortality at 60 days in our study was related to age, functional dependence, the presence of tachypnea or fever, and the use of ceftriaxone. In other studies, age, dementia, frailty, fever, and respiratory symptoms have been associated with higher mortality [7, 8, 12, 21–24]. The relationship between mortality and the use of ceftriaxone, not described in previous studies, could be explained since it is a drug for intravenous or intramuscular administration, which was used in case of inability of oral feeding, therefore assuming a greater severity.

In our study, we did not find differences in mortality at 60 days according to the place of care, data similar to that referred by Bielza [7] or by España, [22] nor its association with male gender or the time of onset of COVID-19 symptoms. The fact of not finding differences in mortality depending on the place of care of the patients, enhances the importance of Liaison geriatric hospital-based team, as key in charge of the comprehensive and individualized assessment of each patient and the most appropriate management, thus avoiding unnecessary admissions and therapeutic fierceness.

The coordination between NH, primary care, and hospital care has been key during the pandemic. A study carried out in France in three nursing homes highlights that those nursing homes that received support from the hospital had lower mortality [25] Furthermore, after the first wave, this new form of work and collaboration has served to implement worldwide new strategies with to improve the care of the older persons living in, such as the formation of liaison geriatric hospital-based team units in our region [9].

The strengths of this study are the large sample size, as well as the multiple variables collected, which have made it possible to obtain results according to previous publications. Moreover, a follow-up of 60 days has been performed, higher than the studies published so far since we consider the impact of the disease on mortality could be better evaluated in a longer period.

The main limitation of our study is that it is retrospective, based on the information recorded in the electronic medical record. Furthermore, not all COVID-19 cases were confirmed by diagnostic test due to the limited access, and only the cases in which the nursing home health professionals requested the assessment of the liaison geriatrician were included. This could mean that we had not detected some older patients who could have been included in the study. Also, we could not collect symptomatic treatment or palliative sedation due to the lack of systematic inclusion in the patient’s medical history. Institutionalized patients did not have advance care directives, so this information could not be used, which on the other hand, would have facilitated decision-making.

## Conclusions and implications

The institutionalized patients evaluated by the liaison geriatric hospital-based team for suspected COVID-19, who were referred to the hospital, presented a better functional and cognitive situation and more frequently received specific treatment for the infection. However, no differences in mortality were found with patients treated in the nursing home. More than a third of the patients had died at 60 days, with higher mortality in those considered the oldest, the most dependent, and those who had presented symptoms such as fever and tachypnea. These findings suggest that the figure of the liaison Geriatrist has been important when individually determining the most appropriate management for each patient.

## Data Availability

The datasets used and/or analyzed during the current study are available from the corresponding author on reasonable request. The manuscript has not been and will not be submitted, in part or entirely, elsewhere for publication and if accepted, the paper will not be published elsewhere.

## Abbreviations

COVID-19: Infection by SARS-CoV-2
NH: Nursing home
FAC: Functional Assessment Classification
GDS: Reisberg’s Global Deterioration Scale for dementia staging
BMI: Body mass index
